# Favourable outcome in a subset of ultra-severe traumatic brain injury patients - a single-centre retrospective study

**DOI:** 10.1016/j.bas.2026.106061

**Published:** 2026-04-17

**Authors:** Alice S. Andersson, Carl Björsten, Niklas Marklund

**Affiliations:** a Department of Clinical Sciences Lund, Neurosurgery, Lund University, Sweden; b Department of Clinical Sciences Lund, Neurosurgery, Lund University and Skane University Hospital, Lund, Sweden

**Keywords:** Traumatic brain injury, Ultra-severe, Pupillary reactivity, Withdrawal of life-sustaining therapy, Neurocritical care, Outcome

## Abstract

**Introduction:**

Ultra-severe traumatic brain injury (us-TBI), defined as Glasgow Coma Scale score (GCS) of five-three, is associated with high mortality and severe morbidity amongst survivors. However, in selected patients a favourable recovery may still be achieved.

**Research question:**

We aimed to characterise what clinical parameters can be used as prognosticators in us-TBI patients.

**Material and method:**

A retrospective, single-centre study of 70 us-TBI patients admitted between the years of 2014-2024. Early clinical, and radiological factors were assessed, and patient outcome (Glasgow outcome scale extended- GOSE) was obtained at 3-12 months.

**Results:**

The median age was 52.5 years, 21 had GCS five on admission, 23 GCS four and 26 had GCS three. Four patients had on admission bilaterally dilated pupils, 35 patients had unilateral mydriasis, 11 had miotic pupils and 20 had normal pupils. Thirty-one patients (44%) succumbed to their injuries. Median GOSE was three, and nine patients (13%) achieved an excellent outcome (GOSE 7-8) - these patients were younger (median age 26 years) and showed normalised pupil reactivity post-operatively. A favourable outcome (GOSE≥5) was achieved in 19 patients (28%). A combination of GCS 3 and bilaterally dilated pupils was uniformly fatal.

**Discussion and conclusion:**

Despite presenting with a low level of consciousness (GCS 3-5) and pupillary abnormalities in 71%, survival was observed in 56% of us-TBI patients, and nine (13%) made an excellent recovery (GOSE 7-8). Improved pupillary reactivity post-intervention may be a positive prognosticator. Our data argue against therapeutic nihilism in us-TBI patient presenting with GCS scores of 5-3.

## Highlights

• The outcome of ultra-severe TBI (us-TBI) is often considered dismal.
• We retrospectively evaluated 70 us-TBI patients, defined as GCS 5-3.
• Of these 56% survived and 13% had an excellent outcome (GOSE 8-7).
• Improved pupillary reactivity postoperatively was associated with a good outcome.
• No patients presenting with bilaterally dilated pupils and GCS 3 survived.

## 1 Introduction

Traumatic brain injury (TBI) is a leading cause of mortality and morbidity globally (Maas et al., 2017). The initial management of severe TBI (most commonly, to date, defined as a Glasgow Coma Scale (GCS) score of 8-3) consists of initial resuscitation including avoidance of hypotension, hypoxia, optional use of osmotic agents to reduce intracranial pressure (ICP), and preferably immediate transport to a level-1-trauma centre. An urgent computed tomography (CT) scan is then followed by mass lesion evacuation and/or decompressive surgery when clinically indicated (Carney et al., 2017). The patient should in addition receive neurocritical care including ICP and cerebral perfusion pressure (CPP) monitoring (Carney et al., 2017; Asgeirsson et al., 1994). Key prognostic factors in severe TBI include patient age, GCS on admission, and pupillary reactivity (Hossain et al., 2023). The mortality in severe TBI varies amongst studies, in part due to heterogeneity in inclusion criteria, and is therefore presented within the wide range of 10-40% (Dawes et al., 2015; Mauritz et al., 2008, 2011; Lan et al., 2020; Hutchinson et al., 2016; Réen et al., 2025; Roozenbeek et al., 2012; Raj et al., 2014). While individual outcomes may be difficult to predict, a variable subset of survivors end up with life-long disabilities affecting both physical and psychosocial aspects of life.

The term ultra-severe TBI (us-TBI) has been used to define patients presenting on admission or post-resuscitation with a GCS score of 5-3 (Majdan et al., 2016; van Dijck et al., 2018; Teasdale and Jennett, 1974). The us-TBI group of patients is heterogeneous, both in terms of patient characteristics such as age and co-morbidities but also vary in their access to neurosurgical treatment and long-term outcome (Rubiano et al., 2015). The decision to proceed with emergency neurosurgical treatment, or to abstain from initiating therapy, raises a number of ethical questions in view of lack of clinical management guidelines, difficulties to address treatment decisions with the family due to time restraints in the emergency setting, and that the patient's own will to survive with substantial deficits is often unknown (Rosenfeld and Mathiesen, 2023). While data on us-TBI patients is still scarce, available literature argues that many patients who do not succumb to their injury in the acute setting will have a long hospitalisation and suffer from significant and often life-long disabilities (Gkantsinikoudis et al., 2024; Chieregato et al., 2017). Thus, whether to initiate treatment remains controversial, not least in the elderly patient. In contrast, premature withdrawal of treatment virtually inevitably results in death, requiring both ethical and resource-demanding considerations (van Dijck et al., 2018; Chieregato et al., 2017; van Veen et al., 2021; Tien et al., 2006). Since prognosis of us-TBI patients has been associated with a uniformly poor prognosis regardless of treatment, a do-not-resuscitate (DNR) decision is common early in the process (Gkantsinikoudis et al., 2024; Chaudhuri et al., 2009), and may lead to treatment nihilism and neurosurgical treatment not being initiated. In particular, old age, and a GCS of three in combination with uni-and/or-bilaterally fixed mydriasis has shown an association with a very high risk of mortality regardless of early management (Chaudhuri et al., 2009; Jahns et al., 2019; Chamoun et al., 2009; Demetriades et al., 2004; Kotwica and Jakubowski, 1995).

However, in most available patient series a small subset of patients achieves a favourable outcome - in some cases even a full recovery - with early, aggressive neurosurgical treatment (Gkantsinikoudis et al., 2024). The factors associated with positive outcome in cohorts of us-TBI patients have not been established, and previous studies are usually small, heterogenous and lacks key information such as post-injury timing and pupillary reactivity (Gkantsinikoudis et al., 2024).

The aims of the present study were to investigate what factors are associated with survival and favourable long-term outcome in us-TBI patients, and to assess what early clinical signs correlate to survival and recoverability in us-TBI patients, including those presenting with bi-or unilaterally fixed and/or dilated pupils.

## 2 Method

### 2.1 Patient selection

We retrospectively investigated the long-term outcome in patients presenting with us-TBI admitted to the Neurosurgical department at Skåne University Hospital in Lund, Sweden, between 2014-2024, identified through the hospital system and ICD codes. Patients aged 15 or older with us-TBI were included. All patients who had a GCS score ≥6 upon arrival to the emergency department or to our neurocritical care unit were excluded, as were those who presented with imminent signs of brain death upon arrival.

Sex, age, American Society of Anaesthesiologists Physical Status (ASA), injury mechanism, hypotonia (defined as a systolic blood pressure <90), desaturation (<90% saturation on pulse oximetry at first assessment) or traumatic cardiac arrest was registered. GCS score at the scene of the accident or upon admission to the emergency department was noted. The last known GCS score before administration of sedation was used and had to be 5-3 for the patient to be included in the study. For inclusion of patients with a GCS of 5-3 at the scene of the injury, no verified improvement upon assessment in the ER could have occurred. The Injury Severity Score (ISS) was noted. Pupillary status was defined as normal, unilaterally dilated, bilaterally dilated, or bilaterally miotic and unresponsive. Improvement of pupillary status was noted after any medical or surgical intervention. Whether the pupils were fixed was also noted.

Administration of osmotic agents preoperatively, such as mannitol, hypertonic saline or urea was noted, as was the use of preoperative hyperventilation. The day of withdrawal of care was noted if such a decision was made. Number of days spent in neurosurgical intensive care unit (NICU) and/or the general ICU, survival at discharge from NICU, the best recorded GCS score during treatment in NICU (Dripps, 1963; Baker et al., 1974; Maas et al., 2005)-days mortality, and whether cause of death was considered intra-or extracranial was gathered. Primary outcome was measured as Glasgow outcome scale extended (GOSE) at 3-12 months post-injury and was extracted from records in patient journals via Melior (Cerner, Kansas City, MO, USA) or by structured follow-up interview by a research nurse (Teasdale et al., 1998).

### 2.2 Imaging

Radiological data was gathered from imaging archives via Sectra Picture Archiving and Communication System IDS7 (Sectra AB, Linköping, Sweden). All CT-scans at admission were analysed according to the Rotterdam CT score and the Marshall TBI classification (Maas et al., 2005; Marshall et al., 1992). Presence of epi-and/or-subdural haematoma, cerebral contusion and traumatic subarachnoid haemorrhage (tSAH) was registered. Haematoma evacuation, insertion of an intracranial pressure monitor (IPM) or external ventricular drainage (EVD), whether the patient had a decompressive craniectomy and time-to-operation from first hospital-assessment measured in hours was registered.

### 2.3 Statistical analysis

Continuous data was assessed using Kolmogorov-Smirnoff test and was found to be non-parametric, therefore expressed by medians and interquartile range (IQ). The Mann–Whitney *U* test was used to compare continuous data between two groups. For comparisons involving more than two groups, the Kruskal–Wallis test was used, and if significant pair-wise comparisons were then performed by Mann–Whitney *U* test. Categorical variables involving two or more groups were analysed using the Chi-squared test. All data was analysed in SPSS (29.0) (IBM Corp, Armonk NY). All data was stored in Microsoft Excel (Microsoft Office, Redmond, WA, USA).

### 2.4 Ethical considerations

The study received approval from the regional ethical review board for the retrospective evaluation of patients (Dnr, 2022-07096-02). The next of kin signed an informed consent and received oral and written information regarding the study and that the patient could be included in a retrospective analysis. Compliance with the General Data Protection Regulation (GDPR) was ensured, and a separate application was submitted for access to medical records (the regional *Kunskapstyrning* (KvB).

## 3 Results

Seventy-one patients were included in the study, but one patient of foreign nationality was excluded due to lack of follow-up information. The median age of the patient population was 52.5 (IQ 27.5-65), 49 were male and 21 were female (70% and 30%, respectively). Twenty-six patients presented with a GCS score of three (37%), 23 as GCS four (33%) and 21 as GCS five (30%). The median ISS was 31.5 (IQ 25-50), and the median ASA score was 1 (IQ 1-2). The most common cause of injury was a motor vehicle-related accident, followed by fall from height over 1 m (Table 1). All patient underwent one or several neurosurgical interventions, except two who were brought to our clinic with intention to treat surgically but due to the extent of progression of their injuries they showed signs of imminent brain death and never received any neurosurgical treatment, and both passed within two days.

**Table 1:** Demographics of the 70 included us-TBI patients.

	Median (IQ) and/or n/(%).range	Missing
Age (years)	52.5(27.5-65), 15-84	-
Gender		-
Female	21 (30%)	
Male	49 (70%)	
GCS at admission	4 (3-5)	-
GCS post-surgery	7 (4-11)	-
Injury mechanism		-
Fall from >1 m	16 (23%)	
Motor vehicle accident	19 (27%)	
Penetrating injury	2 (3%)	
Abuse	6 (8.5%)	
Pedestrian	9 (13%)	
Sports related	2 (3%)	
Fall from <1 m	16 (22.5%)	
Pupillary reactivity on admission		-
Normal	20 (29%)	
Unilaterally dilated	35 (50%)	
Bilaterally dilated	4 (6%)	
Bilaterally miotic	11 (15%)	
Improvement of pupillary reactivity post-surgery	25 (36%)	-
Haematoma evacuation	41 (59%)	-
Haematoma/of which N (%) were evacuated		-
Subdural	44 (63%)/35 (80%)	
Epidural	10 (14%)/8 (80%)	
Contusion	47 (67%)/4 (8%)	
tSAH	56 (80%)	
EVD/IPM	57 (81%)	-
Decompressive craniectomy	14 (20%)	
GOSE	3 (1-5)	1
1 - Dead	31 (45%)	
2 - Unresponsive	3 (4%)	
3 - Lower severe disability	8 (11.5%)	
4 - Upper severe disability	8 (11.5%	
5 - Lower moderate disability	4 (6%)	
6 - Upper moderate disability	6 (9%)	
7 - Lower good recovery	5 (7%)	
8 - Upper good recovery	4 (6%)	
Time of GOSE assessment amongst survivors	12 (6-12)	
Time spent in NICU	7 (4.5-16.25)	-
Hypotension on admission	10 (14%)	-
Hypoxia on admission	14 (20%)	-
Pre-hospital cardiac arrest	4 (6%)	-
Pre-surgery administration of mannitol/Urea/Hypertonic saline	37 (53%)	
Hyperventilated	26 (37%)	
ASA score	1 (1-2)	-
ISS	31.5 (25-50)	-
Rotterdam CT score of traumatic brain injury	4 (3-5)	-
Marshall classification of traumatic brain injury	3.5 (2-4)	-
Withdrawal of care in days (n = 25, 36%)	2 (1-4)	-
Time to OR (hours from admission)	3 (1-5)	-
Death within 30-days (% of those who died)	31 (100%)	-
Cause of death (n = 31)		-
Intracranial	29 (94%)	
Extracranial	2 (6%)	

Glasgow coma scale (GCS), External ventricular drains (EVD), Intraparenchymal pressure monitor (IPM), Glasgow outcome scale extended (GOSE), Neurosurgical intensive care unit (NICU), American society for anaesthesiology (ASA), Injury severity score (ISS), Computer tomography (CT), operating room (OR). Improvement of pupillary status was noted after medical or surgical intervention, defined according to an ordinal scale where one dilated pupil is superior to two, and normal pupils superior to one dilated pupil.

Cerebral contusions and subdural hematomas (SDH) were the most common intracranial injuries (n = 47, 67% and n = 44, 63%, respectively), resulting in four contusion evacuations and 35 SDH evacuations. Ten patients had epidural haematomas (EDH) of which eight were evacuated (Table 1). The median Marshall classification of traumatic brain injury score was 3.5 (IQ 2-4), and the median Rotterdam CT score of traumatic brain injury was 4 (IQ 3-5)(Table 1). The median time from initial assessment to surgical intervention was 3 h (IQ 1-5), and 57 patients received an IPM or an EVD (81%) and 41 underwent evacuation of EDH, SDH, contusions or a combination thereof (59%). A decompressive craniectomy was performed in 14 cases (20%). Administration of osmotic agents was performed in 52% (n = 37) of cases and 37% (n = 26) were hyperventilated preoperatively. The median number of days spent in NICU was seven days (IQ 4.5-16.25) (Table 1).

The median GOSE score was 3 (IQ 1-5). GOSE could be obtained from all patients except one. This patient is alive, but his functional level could not be assessed. Nine/69 patients (13%) had an excellent recovery (GOSE seven and eight), whereas 19 patients (28%) had a favourable outcome (defined as a GOSE ≥5). Thirty-one patients succumbed to their injuries (45%), all within 30 days post-injury, and cause of death was attributed to their intracranial injury in 29 (94%) of the patients. Withdrawal of care was decided in 25 cases (36%), at a median time of two days after admission (IQR 1-4). Of patients requiring surgical evacuation, 17 died after SDH evacuation, three after EDH evacuation, and two after contusion evacuation.

### 3.1 Glasgow coma scale-3 (Fig. 1a)

**Fig. 1:**
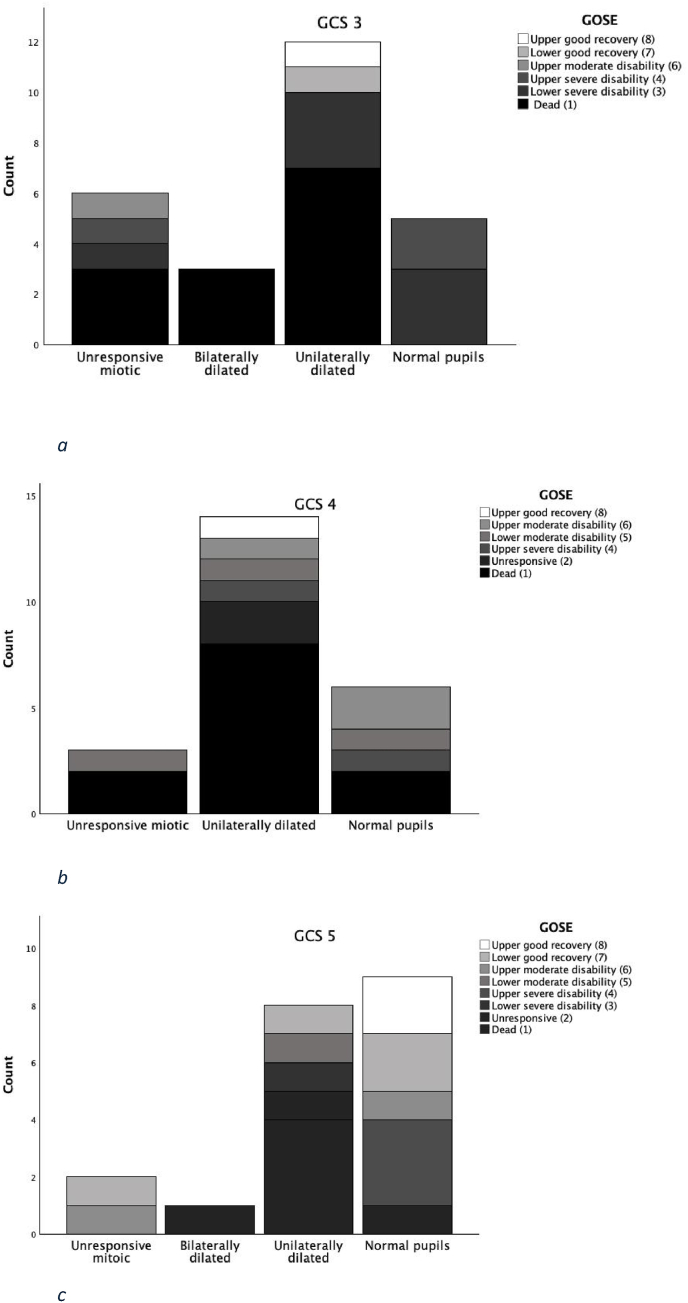
Bar graphs showing the distribution of outcome according to Glasgow outcome scale extended (GOSE) in patient groups categorized by Glasgow coma scale (GCS) five to three, based on patient pupillary status. Fig. 1a) demonstrates the outcome in patients presenting as GCS 3, Fig. 1b) the outcome in patients presenting as GCS 4, and Fig. 1c) the outcome in patients presenting as GCS 5.

Twenty-six patients presented as GCS 3. The median age was 50 years (IQR 36.25-59.25). The median ASA score was 1 (IQR 1-2), and the median ISS was 35 (IQR 25-60.25). Twenty-two patients received an IPM, and seven had an emergent craniotomy/craniectomy performed. The median time spent in the NICU was nine days (IQ 2.75-20). The median GOSE was 2 (IQR 1-3). Three out of the 26 patients that presented with a GCS score of three had bilaterally dilated pupils at admission, of whom none were alive at GOSE evaluation. Twelve had a unilaterally fixed, dilated pupil of whom seven died and three had a GOSE score of three (Fig. 1a). Of all, 13 patients died (50%), 7 had a GOSE score of three (27%) and only one had a GOSE score of eight - a 60-year-old patient presented with a unilaterally dilated pupil and received an emergent craniotomy for an isolated acute subdural haematoma within 1 h of presentation.

### 3.2 Glasgow coma scale-4 (Fig. 1b)

Twenty-three patients presented as GCS four, of whom 12 died (52%), two were unresponsive (GOSE 2) at long-term follow up, and one had a GOSE score of eight, making an excellent recovery. This 25-year-old multi-trauma patient with an ISS score of 45 had a traumatic subarachnoid haemorrhage and a subdural haematoma that was immediately evacuated. The patient also demonstrated an improvement of his pupillary status - from one dilated pupil to normal pupils after medical and surgical intervention. The median GOSE in this group was 1 (Maas et al., 2017; Carney et al., 2017; Asgeirsson et al., 1994; Hossain et al., 2023; Dawes et al., 2015), and the median age in this group was 54 years (IQR 25-70). Fourteen patients had a unilaterally dilated pupil, and none presented with bilaterally dilated pupils (Fig. 1b). Fifteen patients underwent surgical evacuation of an intracranial haematoma, four had a craniectomy and 18 received an IPM. The median ASA score was 2 (IQR 1-3), and the median ISS was 26 (25-50). The median time spent in NICU was 7 days (IQR 5-13).

### 3.3 Glasgow coma scale-5 (Fig. 1c)

Twenty-one patients presented as GCS five, of whom six died (29 %), four patients had a GOSE score of 7 and two had a GOSE score of 8 3-12 months post-injury. The median GOSE was 4 (IQR 1-7). The median age was 55 years (IQR 26-68.5). Eleven patients underwent haematoma evacuation surgery, three had a craniectomy and 17 received an IPM. The median ASA score was 2 (IQ 1-2.5), and the median ISS was 30 (IQ 25-43). Only one had bilaterally dilated pupils, and nine had a unilaterally dilated pupil (Fig. 1c). The median time spent in NICU was 11 (3.5-16). Patients presenting as GCS five upon admission had a significantly higher median GOSE score at 3-12 month follow up than those presenting as GCS three (GOSE four and two, n = 21and n = 26, respectively; p < 0.01).

### 3.4 Pupillary status

Most commonly, us-TBI patients presented with a unilaterally dilated pupil (n = 35, 50%). Twenty patients had normally reacting pupils (29%), four patients had bilaterally dilated (6%), and 11 patients had bilaterally miotic, unresponsive pupils (16%). Of the patients with pupillary abnormalities, 24 (48%) had an improvement in their pupillary status after initial resuscitation and surgical intervention. Patients with pupillary abnormalities suffered a worse outcome when compared to patients with normal pupils (median GOSE one vs. four, respectively; p < 0.05) and had higher Rotterdam CT scores (4 and 3, respectively; p < 0.001). Patients who had an improved pupillary status after initial resuscitation and/or surgical intervention had a better prognosis when compared to those who did not experience pupillary improvement (median GOSE of three versus two, IQR 1-5, respectively).

### 3.5 Which us-TBI patients had a good outcome?

**Fig. 2:**
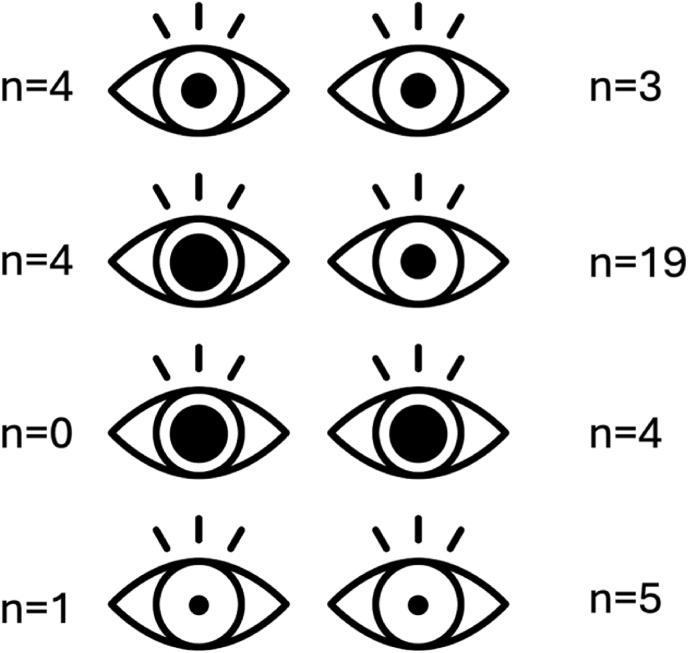
Illustration presenting the differences in pupils between patients with a full recovery, defined as Glasgow outcome scale extended (GOSE) 7-8, to patients who passed due to their injury (GOSE 1).

Nine of the us-TBI patients in our present cohort had a GOSE score of 7-8 (Table 2). Six patients presented as GCS five, one as GCS four and two as GCS three. None suffered any prolonged hypotension, hypoxia or cardiac arrest. Four of these patients had normally reactive pupils (44%), four had a unilaterally dilated pupil (44%) and one presented with unresponsive miotic pupils (11%) (Fig. 2). None had bilaterally fixed and dilated pupils. Four of those with pupil abnormalities demonstrated improvement in pupillary status after medical and/or surgical intervention (80%). The median time to surgical intervention was 3 h (IQR 1-5); Three patients underwent subdural haematoma evacuation, and eight received an IPM. No one received a craniectomy, four received hyperosmolar treatment in the preoperative acute setting and three were hyperventilated preoperatively. The median Marshall classification score was three (IQR 2.5-4), and the median Rotterdam CT score of TBI was 4 (IQR 3-4). Examples of CT scans of patients with a) good outcomes and b) poor outcomes are shown in Fig. 3a, Fig. 3b.

**Table 2:** Differences and similarities between good recovery us-TBI patients, defined as Glasgow outcome scale extended (GOSE) 7-8, and us-TBI patients that succumbed to their injuries (GOSE 1). American Society of Anaesthesiologists Physical Status (ASA), Injury severity score (ISS), Glasgow coma scale (GCS), Subdural haematoma (SDH), traumatic subarachnoid haemorrhage (tSAH).

	GOSE 7-8	GOSE 1	P-value
Number of patients	9	31	*-*
Median age	26 (21-61.5)	56 (44-65)	*0.208*
Median ASA score	1 (1-2)	2 (1-3)	*0.318*
Median ISS	26 (25-43)	26 (25-43)	*0.736*
Median GCS	5 (3.5-5)	4 (3-4)	***0.021***
Most common lesions	SDH & tSAH	SDH & tSAH	-

**Fig. 3a:**
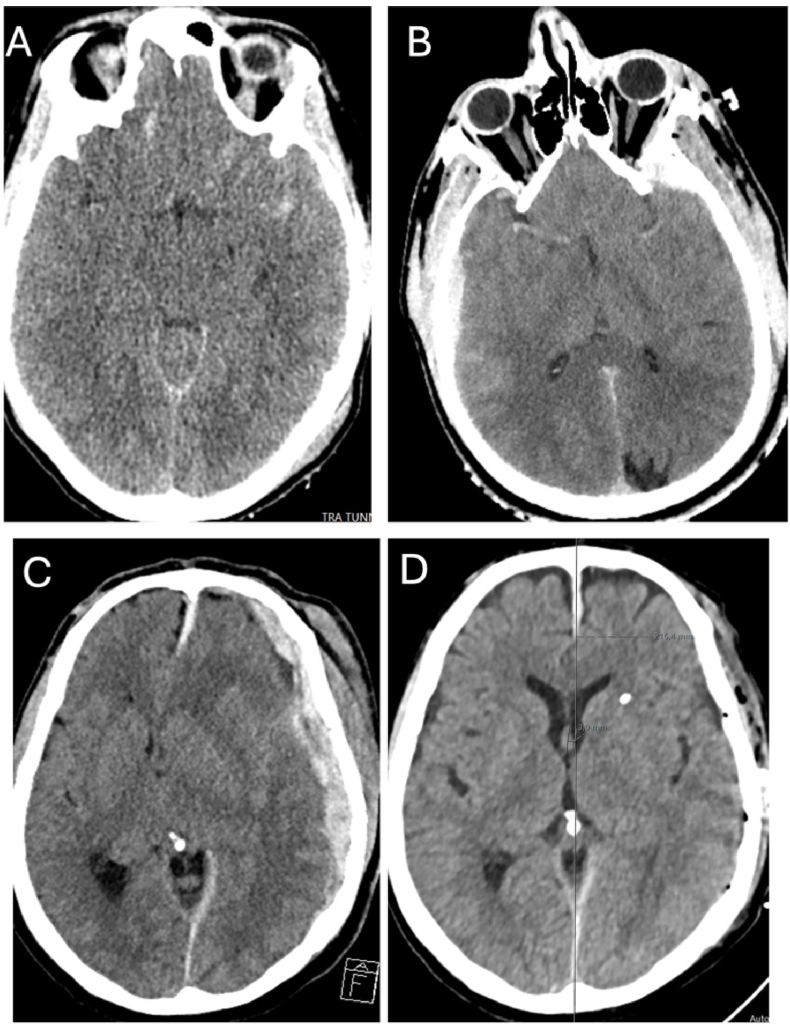
CT scans of us-TBI patients achieving a good outcome (Glasgow outcome scale extended, GOSE, 7-8). In A), a 19-years old female was found 10m from her car after a motor vehicle accident at an approximate speed of 90 km/h. On assessment, she was GCS 5 with bilaterally small unresponsive pupils. On CT she had signs of widespread traumatic axonal injury. She had an uneventful neurocritical care course, received a tracheostomy and achieved an excellent recovery after a lengthy neurorehabilitation. B) A 26-years old male involved in a motorcycle accident and was GCS 5at the scene of the accident. He had on CT bilateral epidural hematomas and a complex fracture system involving the bilateral skull base, orbital fractures, bilateral temporal bone fractures and a zygomatic arch fracture on the left side. He received ICP monitoring and wound revision and could be extubated on day 3. C-D) A 60-year-old male was beaten and found outside at a GCS of 9-10. At the ER of the local hospital, 20 min away from our neurosurgical department, he rapidly deteriorated to GCS 3 and dilated left pupil. He was taken to our department for emergent surgery and evacuation of the left-sided acute subdural haematoma. Already at one day post-surgery (D) he was GCS 14-15, with normal pupils and could be extubated.

**Fig. 3b:**
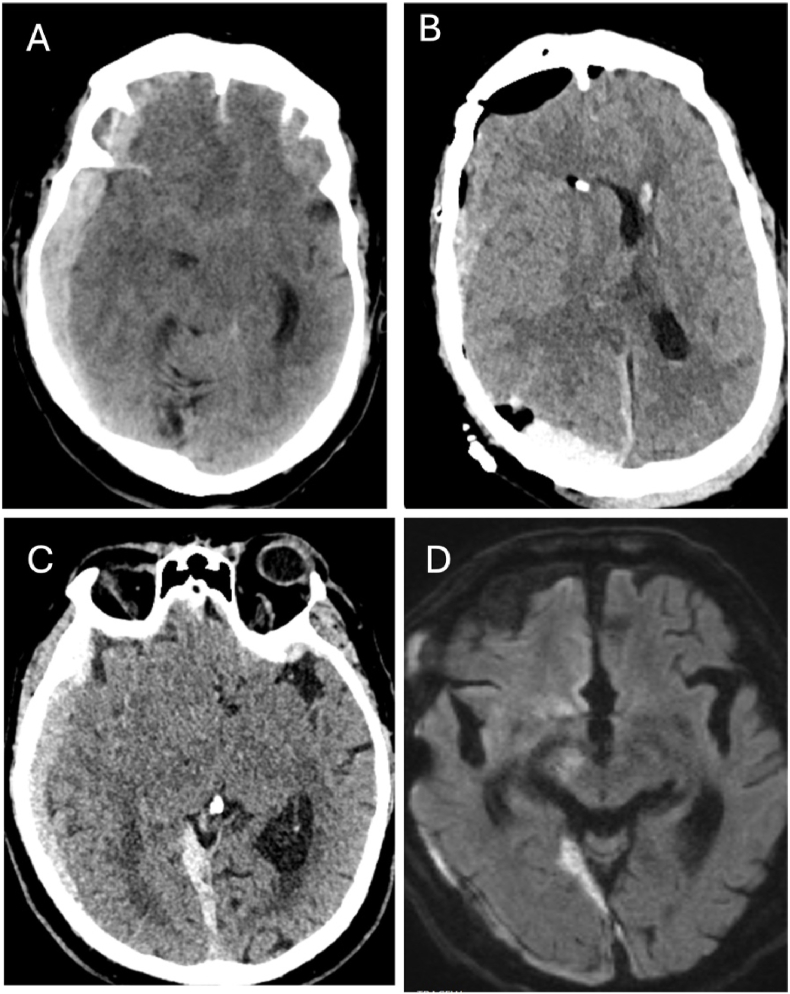
Examples of CT scans of us-TBI patients with a fatal outcome (Glasgow outcome scale extended, GOSE, 1). A-B) A 60-year-old male with a fall accident at work. At the ER he was initially awake, then rapidly deteriorated to GCS 4 with bilaterally dilated but responsive pupils at the local hospital ca 2 h away from our neurosurgical department. Upon arrival, he was immediately taken to surgery for removal of the acute right-sided subdural haematoma. Post-op CT scan (B) shows evacuation of the haematoma although a persistent midline shift and right-sided cerebral swelling was observed. He was then GCS 4 with bilaterally unresponsive pupils. A do-not-resuscitate order was taken on post-op day 2, and step-down care after day 4. He died soon thereafter. C-D) A 76-year-old male on warfarin due to atrial fibrillation fell and deteriorated rapidly to GCS 4 and dilated his right pupil. The warfarin effect was immediately reversed, and the patient was taken to our department where he underwent emergent craniotomy and evacuation of the haematoma. The post-operative CT scan showed a resolution of the midline shift. However, the patient was post-operatively GCS 6 which persisted. An MRI (D) ca 2 weeks post-surgery shows right-sided central infarcts and withdrawal of care was decided on day 20 post-op.

Of the 31 patients that succumbed to their TBI, nine patients were hypoxic before resuscitation, five were hypotensive and three had a traumatic cardiac arrest (Table 2). Most patients who died had a unilaterally dilated pupil at presentation (n = 19, 61%), and four had bilaterally dilated pupils (Fig. 2, legends). Ten patients demonstrated an improvement in pupillary status post initial resuscitation (32%). The median time to surgical intervention was 2.5 h (IQ 1-5.25). Twenty-three patients underwent haematoma evacuation, 22 patients got an IPM, and nine patients had a craniectomy.

### 3.6 Withdrawal of care

A decision to withdraw care was made in 25 patients, at a median time of two days post-injury (IQR 1-4). The median age amongst those 25 patients was 61 (IQR 45-66) years, the median admission GCS was 4 (IQR 3-4), of whom only one presented with a normal pupillary status. The majority had one dilated pupil (n = 17, 68%), two patients had bilaterally dilated (8%) and five patients had bilaterally miotic pupils (20%). The median ISS was 26 (25-43). Evacuation of intracranial haematoma was performed in 19 cases (76%), and six patients underwent a decompressive craniectomy (24%). Two patients had an episode of pre-hospital cardiac arrest or upon arrival to the emergency department (8%), four patients were circulatory unstable upon arrival (16%) and six were hypoxic upon arrival to the emergency department (24%). All patients in this group died due to their intracranial injuries. Withdrawal of care was done within 24 h of admission in nine patients - none of these had normal pupils at first assessment, seven had a unilateral pupil dilation and six patients underwent haematoma evacuation.

## 4 Discussion

In this retrospective single-centre study our aim was to investigate the factors associated with survival and good long-term outcome (GOSE 7-8) in us-TBI patients, defined as a Glasgow Coma Scale score of 5-3. The major findings of our present study were that, although a minority, some us-TBI patients do make an excellent recovery and what characterises them is their young age, lack of serious systemic co-morbidities and lack of bilaterally fixed pupils (Table 2). Moreover, improvement in pupillary status was associated with survival and a potential for favourable outcome. This is in line with earlier studies, since improvement of pupillary status has been linked to a higher chance of positive outcome, emphasizing the value of continuous pupillary control, especially after first assessment (e.g. administration of hypertonic saline, mannitol or hyperventilation) and surgical intervention (Monai et al., 2023). In the group of patients with pupillary abnormalities there was a higher Rotterdam CT score when compared to patients with normal pupils (4 vs. 3), which has been shown to correlate with patient outcome (Maas et al., 2005; Charry et al., 2017).

The use of outcome measures differs among TBI studies. By choosing to focus on those who make a near to-or-total recovery (GOSE 7-8) we wanted to emphasize that it is possible to make a near-full recovery after sustaining an us-TBI, avoiding cultural-and-or individual opinions on the definition of a life worth living. A GOSE 5 and 6 is often used as a definition of a favourable outcome in TBI studies, and was achieved by 19 (28%) of the us-TBI patients included in our study.

Perhaps not surprising, also in us-TBI patients was high age and low GCS score associated with a low likelihood for a good outcome. In particular, a GCS score of three in combination with bilaterally, unresponsive, dilated pupils was uniformly fatal (Gkantsinikoudis et al., 2024; Tien et al., 2006). Outcome was also better in the GCS 5 cohort when compared to those in GCS 3. We also observed that - despite similar median ISS scores – that the median age in the group with a long-term outcome of GOSE 7-8 was lower when compared to the age of those who passed (median 26 years *vs.* 56 years, respectively).

On neuroimaging, there was an expected distribution of EDHs, SDHs and cerebral contusions within the studied population. SDHs were the most frequently surgically evacuated lesion (n = 36), although with less than half of patients operated surviving. Three out of eight EDH patients were dead on outcome assessment, demonstrating a somewhat better survival in this group.

Withdrawal of care was done in a median time of the second day of admission, and in nine patients already on the first day. Withdrawal of care was observed in 81% of fatal us-TBI cases in our cohort. The reasons for this varied, but most often due to poor neurological state post-operatively. Investigation by MRI was not consistently used prior to the decision of withdrawal of care, instead most often used when there was a suspicion of traumatic axonal injury. While there are cultural and/or individual differences in what is considered a worthy survival, there is usually consensus amongst health-care providers that an unresponsive state is an unfortunate outcome (Allen et al., 2023). Therefore, in this group of patients, there is definite value in defining both positive and negative prognosticators. These early decisions to withdraw care do not align with several studies suggesting at least 48 h and preferably 72 h of observation before deciding whether to terminate active care (van Veen et al., 2021; Souter et al., 2015).

The median time of GOSE assessment was most often performed at 6-12 months post-injury. Long-term (> one year) follow-up studies in this group of us-TBI patients is lacking. It may also be possible that the number of patients with a fortunate outcome (GOSE 7 or 8) would be higher in our cohort, given a longer follow-up period. Our present data may therefore be a pessimistic representation of the long-term, positive outcome since social and cognitive qualities may improve over a longer period, resulting in a greater independence for the patients. In the RESCUE-ICP trial, patient outcome had improved at two-years postinjury when compared to the earlier, 6-month evaluation (Hutchinson et al., 2016). The outcome may also be affected by treatment bias, i.e., a patient may receive less treatment intensity than others due to e.g., high age or co-morbidity, in view of the well-known association with these prognostic factors. The presence of treatment nihilism, and self-fulling prophecies will inevitably skew the results and potentially lead to a worse outcome in study cohorts (Gkantsinikoudis et al., 2024).

Our study is not without limitations. This study is prone to selection bias since it only includes patients who has been admitted to our neurosurgical department. Patients severely ill arriving to a level II trauma centre, where the time of transportation is simply too long may have resulted in the decision to abstain from transport to our clinic and such patients are therefore not included. No wake-up assessments were performed routinely prior to surgery, and patients stabilized at a level II trauma centre were intubated before transport to us. Thus, GCS assessment was not performed by a neurosurgeon, although often accompanied by a clinical description of neurological status by the emergency physician at the primary, local hospital. We observed that a relatively large subset of us-TBI patients achieved a favourable outcome. We cannot exclude the possibility that some patients assessed as us-TBI had a relatively less severe injury. Pre-hospital sedation and post-injury seizures may make the GCS assessment less reliable both at the pre-hospital and ER setting. We want to emphasize, however, that the GCS was in most cases reported the trauma scene, and most often remained consistent with first described GCS score upon arrival at the hospital. The study's strengths lay in its access to highly detailed clinical data, and close-to-none missing data apart for one surviving patient's clinical status where GOSE details could not be obtained.

## 5 Conclusion

In this retrospective study investigating the factors associated with survival and positive long-term outcome in us-TBI patients we found nine (13%) patients reaching a good outcome, and 39 (56%) patients survived. We found improvement in pupillary status as a possible positive clinical prognostic factor for a good outcome. We also emphasize the importance of a sufficient observation time (e.g. 48-72 h) to evaluate potential clinical improvement. The prognosis of patients presenting with a GCS of three in combination with bilateral pupil dilation was dismal. We further show that it is possible for patients to make an excellent recovery after experiencing us-TBI, and further studies should be conducted to improve our ability to identify those with a potential for reaching a favourable recovery.

## Author contributions

All authors contributed to the study conception and design. Material preparation, data collection and analysis were performed by Alice Andersson and Carl Björsten. The primary draft of the manuscript was written by Alice Andersson and Niklas Marklund. The study was supervised by Niklas Marklund, and he also designed the methodology. All authors read and approved the final manuscript before submission.

## Competing interests

Niklas Marklund is European editor of Journal Neurotrauma. No other author declare any conflict of interest.
